# Audiovisual Interval Size Estimation Is Associated with Early Musical Training

**DOI:** 10.1371/journal.pone.0163589

**Published:** 2016-10-19

**Authors:** Mary Kathryn Abel, H. Charles Li, Frank A. Russo, Gottfried Schlaug, Psyche Loui

**Affiliations:** 1 Harvard College, Cambridge, Massachusetts, United States of America; 2 Beth Israel Deaconess Medical Center and Harvard Medical School, Boston, Massachusetts, United States of America; 3 Ryerson University, Toronto, Canada; 4 Wesleyan University, Middletown, Connecticut, United States of America; Universidad de Salamanca, SPAIN

## Abstract

Although pitch is a fundamental attribute of auditory perception, substantial individual differences exist in our ability to perceive differences in pitch. Little is known about how these individual differences in the auditory modality might affect crossmodal processes such as audiovisual perception. In this study, we asked whether individual differences in pitch perception might affect audiovisual perception, as it relates to age of onset and number of years of musical training. Fifty-seven subjects made subjective ratings of interval size when given point-light displays of audio, visual, and audiovisual stimuli of sung intervals. Audiovisual stimuli were divided into congruent and incongruent (audiovisual-mismatched) stimuli. Participants’ ratings correlated strongly with interval size in audio-only, visual-only, and audiovisual-congruent conditions. In the audiovisual-incongruent condition, ratings correlated more with audio than with visual stimuli, particularly for subjects who had better pitch perception abilities and higher nonverbal IQ scores. To further investigate the effects of age of onset and length of musical training, subjects were divided into musically trained and untrained groups. Results showed that among subjects with musical training, the degree to which participants’ ratings correlated with auditory interval size during incongruent audiovisual perception was correlated with both nonverbal IQ and age of onset of musical training. After partialing out nonverbal IQ, pitch discrimination thresholds were no longer associated with incongruent audio scores, whereas age of onset of musical training remained associated with incongruent audio scores. These findings invite future research on the developmental effects of musical training, particularly those relating to the process of audiovisual perception.

## Introduction

Pitch is a subjective attribute related to the fundamental frequency of sound, and the perception of pitch plays an important role in music, speech, and auditory scene analysis. Although our sense of pitch is usually well developed even without formal instruction, there are substantial individual differences in human beings’ sensitivity to pitch; for instance, people with congenital amusia (tone-deafness) have difficulty perceiving differences in pitch less than one semitone apart [1,2]. Even amongst individuals without tone-deafness, substantial individual differences in pitch interval perception are observed [3]. These individual differences are related to, but not completely predicted by musical training, and may be related to linguistic background and individual differences in pitch perception in speech and language [4,5].

Given the wide range of individual differences in pitch perception, it is reasonable to inquire as to how these individual differences may affect the many domains of everyday life that require pitch processing. Influences of individual differences in pitch perception have been observed in speech and language processing [6], spatial processing [7] (but see [8]), and working memory tasks [9]. These findings suggest that difficulties in pitch perception are not isolated deficits, but may affect a network of perceptual and cognitive functions not necessarily unique to music.

The existence of these individual differences suggests that people with low pitch perception or discrimination capabilities may have to develop some compensatory neurocognitive mechanisms in order to cope with domains of everyday life that typically require pitch perception, such as auditory scene analysis and speech processing. One possible compensatory neurocognitive mechanism could be an increased reliance on visual information during the processing of vocal communication, including but not limited to speech. If such a mechanism exists, it could be fruitful to examine how individual differences in pitch perception might relate to multisensory perception.

A previous experimental paradigm for investigating the use of visual cues in pitch perception comes from [10], who sought to determine whether singers’ facial expressions and head movements convey information about pitch intervals that can be “read” by viewers. Trained vocalists were instructed to sing numerous melodic intervals and were filmed during the process. Subjects were then played these visual recordings without sound and were asked to rate the interval size that they imagined each performer was singing, using only visual information. On average, subjects (who had between zero and eight years of musical training) were able to differentiate interval size based only on facial expression and head movement, indicating that viewers rely on visual as well as audio information to process interval size. From this experiment and others using various audiovisual paradigms [11,12,13], it is clear that even in a presumed auditory task, human subjects tend to take into account simultaneously presented visual information.

In the present study, our goal was to determine how individual differences in pitch perception relate to the perception of audiovisual stimuli, as moderated by factors including age of onset and number of years of musical training. An audiovisual experiment was administered to subjects with a range in pitch perception abilities and levels of musical training. To encourage responses based on perceptual information, and to discourage listeners with higher levels of musical training from referring to categorical knowledge, we adopted procedures for interval size judgment developed by Russo and Thompson (2005) [14], with point light displays of singers producing pitch intervals. The use of point light displays allows us to abstract biological movement [15] independent of static facial cues or other visual triggers. Compared to full videos of singers, the use of point light displays eliminates possible confounds of visual memory for any other visual cues (such as static facial cues that might be specific to certain intervals), thus limiting our visual cues to biological movement.

## Methods

### Participants

A total of fifty-seven subjects participated in the study. Participants completed the experiment in return for course credit at Wesleyan University (n = 36) or were recruited through online advertisements in the Boston area (n = 21). The advertisements were meant to attract individuals with a range of musical experience and expertise. All subjects provided written informed consent, which was approved by the Institutional Review Board of Beth Israel Deaconess Medical Center or by the Psychology Ethics Board of Wesleyan University.

All subjects had normal hearing by self-report and were right-handed as determined using the Edinburgh Handedness Inventory [16]. The sample consisted of 36 females and 21 males, with an age range from 18 to 52 years (mean = 22.64 years, SD = 6.86). Surveys were administered to all subjects to assess their linguistic and musical backgrounds, specifically whether they had received musical training and, if they had, what age they began training, how long they received training, and on what instrument. Thirty-two of all subjects reported having had musical training, with an average of 6.84 years (SD = 4.18, Range = 1 year to 13 years) in a range of instruments including piano (n = 18), violin (n = 10), guitar (n = 8), flute (n = 3), cello (n = 2), drums (n = 2), and oboe, trumpet, voice, and recorder (n = 1, each). Some subjects played multiple instruments; thus, instrument numbers as listed include overlap between subjects. Age of onset of musical training ranged from 5 to 15 years of age (mean = 8, SD = 2.62). There were no significant differences between the Wesleyan subjects and the Boston subjects in age of onset or number of years of musical training (age of onset: t(30) = 1.4, p = .17, number of years: t(30) = 1.88, p = .07); thus the two groups were combined for all subsequent analyses.

Shipley abstraction test was also given as a self-administered task designed to measure intellectual functioning [17]. It is a pattern-completion task consisting of 20 items with strings of letters or numbers, in which subjects are asked to complete the pattern by filling in letters or numbers, e.g. AB BC CD D- (target answer: E). Performance on the Shipley abstraction test has been shown to correlate highly with nonverbal IQ [18]. The average abstraction test score in our sample was 16.8 (SD = 1.7).

Pitch discrimination ability was tested using a psychophysical staircase procedure (three-up, one-down) centering at a frequency of 500 Hz [19] to calculate a pitch perception threshold. In the pitch perception test, subjects hear pairs of tones and are asked to judge whether the second tone is higher or lower in pitch than the first. The test averages the pitch frequency differences at the first six turnaround points, thereby yielding a pitch discrimination threshold. The average pitch discrimination score was 13.0 Hz (SD = 16.0), with scores ranging from 0.8 to 68.0 Hz. For further analyses, pitch discrimination thresholds were log-transformed to achieve a normal distribution.

### Stimuli

The stimuli consisted of videos of three female vocalists, who sang 13 ascending melodic intervals spanning 0 to 12 semitones (from [11]). Each singer heard the target interval through headphones, then repeated the interval using a “la” syllable. The singers attempted to match the pitch and duration (approximately 1.5 seconds per tone) of the target interval to standardize across all trials. Each interval was in a comfortable range for the three singers. The x- and y- coordinates of passive markers placed on the nose (showing head movement), the top of each eyebrow, the upper edge of the upper lip, and the lower edge of the lower lip were all tracked using Adobe After Effects CS3 (Track Type: Transform/Position, Channel: RGB, Subpixel Positioning: On). This tracking system creates a point-light image, in which only the markers are illuminated and the rest of the face is not visible. Using iMovie, three different alterations of the stimuli were created: audio-visual stimuli (with sound and video combined), audio-only stimuli (with sound and no video), and visual-only stimuli (with video and no sound) of the sung intervals. Of the audio-visual stimuli, both “congruent” and “incongruent” trials were created. For the “congruent” trials, the visual and auditory stimuli were not changed (i.e., the sung interval matched the corresponding visually seen interval); in contrast, the visual and auditory stimuli were mismatched in the “incongruent” trials. For example, in the “incongruent” trial, the auditory stimulus would be a sung octave, but the visual stimulus would be the facial movements corresponding to the interval of a fifth. Of note, the incongruous auditory and visual stimuli were paired together for each singer; auditory and visual stimuli were never mixed between different singers. A total of 180 incongruent audio-visual trials were created by matching each visual stimulus (13 ascending intervals x 3 singers = 39 visual stimuli) with intervals of 0, 3, 6, 9, and 12 semitones (39 visual stimuli x 5 intervals = 195 incongruent audio-visual trials, but the 15 videos that were congruent with respect to the auditory and visual stimuli were removed from the incongruent set).

### Procedure

Max/MSP [20] was used for trial presentation and data collection. Testing was done in a quiet testing room at BIDMC, and in a comparable quiet testing room at Wesleyan. Auditory stimuli were presented at approximately 70 dB SPL, as calibrated using a sound level meter in each testing environment. In total, the experiment consisted of 39 audio-only trials (Batch A), 39 visual-only trials (Batch V), 39 congruent audio-visual trials (Batch C), and 180 incongruent audio-visual trials (Batch I). The 180 incongruent audio-visual trials were randomly divided into three groups of 60 trials. Subjects rated each trial in Batches A, V, and C three times and each trial in Batch I once. This design was employed to ensure that each subject rated each trial at least once. One “run” consisted of Batch A, Batch V, Batch C, and a randomly selected one of the three groups from Batch I. The second run consisted of Batch A, Batch V, Batch C, and one of the two remaining randomly selected groups from Batch I. The final run consisted of the three recurring batches, as well as the final group from Batch I. The order of trials within each batch, the order of batches within each run, and the order of runs were all randomized. After each individual trial, the subjects used a sliding visual analog scale (controlled by the computer mouse) to rate the size of the interval, using all of the information available to them including pitch, facial expression, and movement of the mouth. The slider height was continuous but was rounded to the nearest integer ranging from 1 (indicating a small interval) to 12 (indicating a large interval) in our experiment software. The sliding visual scale was chosen to allow subjects who did not have explicit knowledge of interval size (e.g. nonmusicians) to still make ratings for interval size (i.e. instead of thinking that an interval was e.g. “a 5 out of 12” they could simply indicate that it was a moderate-sized interval). Subjects were asked to judge the pitch interval, using all of the information available to them. A practice run was conducted with one trial of each trial type before the proper experiment, to ensure that subjects understood the instructions as well as the mapping between interval size and the visual analog scale. Between each run, subjects were allowed to pause and take a break before returning to the computer. The experiment lasted a total of approximately 45 minutes.

### Data analysis

For each subject, interval ratings were averaged across the three trials for each interval for the A, V, and C condition, respectively. In the I condition, subjects received only 1 trial per interval, and so no averaging was performed. Ratings from all four conditions were then correlated with the actual interval size to obtain a measure of accuracy. Correlations between stimulus and rating for A, V, and C conditions were computed for each subject, resulting in a possible score ranging from -1 (the improbable case of a perfectly inverse correlation between stimulus and rating) and +1 (perfect correlation between stimulus and rating) for each condition for each subject. Since audio and visual stimuli were mismatched in the I condition, this mismatch provided an opportunity to estimate the degree to which subjects used audio versus visual information in their rating. The correlation between veridical interval size of the audio interval (in semitones) stimulus and rating was computed as an incongruent audio score (the AV-I:A score), and correlation between veridical interval size of the visual interval and rating was computed as an incongruent visual score (the AV-I:V score). This resulted in an incongruent audio score and an incongruent visual score (each ranging again from -1 to +1), which indicated the degree to which each subject’s ratings reflected auditory or visual information when faced with incongruent audiovisual information.

## Results

We first used Pearson correlations from the A, V, and C conditions to determine how participants’ ratings correlated with the veridical interval size. In the A condition, participants’ ratings were highly correlated with the veridical interval size, as shown by the high average correlation score (*r* = 0.90, *p* < 0.0001). Participants’ ratings were also significantly correlated with the actual interval size in the V condition (*r* = 0.60, *p* < 0.0001). Results of the V condition corroborate previous studies on judgments of visual interval size [10,21]. Ratings in the C condition were also significantly correlated with the actual interval size as seen and heard in the stimulus (*r* = 0.889, *p* < 0.001).

As a difference in performance between C and A conditions provides evidence for the influence of visual information, we computed the difference between correlations for the C condition and the A condition to obtain a difference score that represents the extent to which participants were affected by visual information over and above auditory information. The average of this difference score was -0.013 (SD = 0.069), suggesting that participants were on average slightly more likely to be distracted by visual information in the audiovisual congruent condition (with higher r values for A than for C), although the overall effect was not statistically significant and participants were variable. Interestingly, however, this difference score was significantly correlated with Shipley score (*r* = 0.53, p < 0.01): those with higher Shipley scores had more positive values in the audiovisual congruent condition, indicating more contribution of visual information. This suggests that subjects who had higher nonverbal IQ could have been more likely to use visual information over and above auditory information when audiovisual stimuli were both present. This was confirmed by comparing correlation scores in the V condition between high-Shipley and low-Shipley scorers (categorized by a median split). High-Shipley subjects’ ratings showed significantly higher correlation coefficients with the visual stimuli compared to low-Shipley subjects (high-Shipley scorers: r = 0.67 ± 0.023 vs. low-Shipley scorers: r = 0.52 ± 0.053, t(55) = 2.28, p = 0.026).

**Fig 1:**
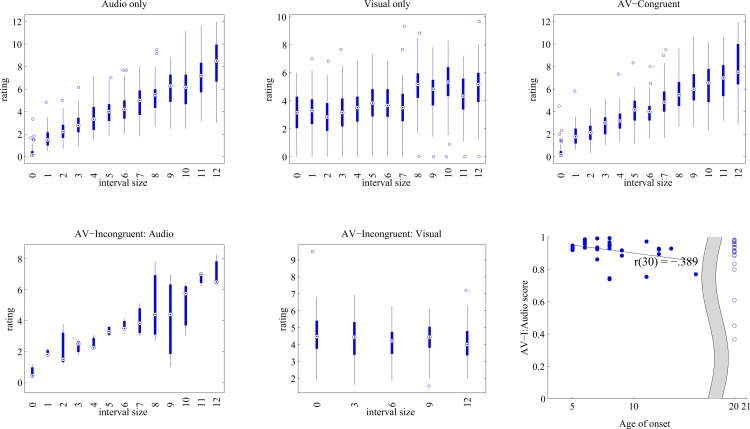
Interval size ratings results from Audio (A), Visual (V), Audiovisual-Congruent (C), and Audiovisual-Incongruent (I) conditions, and relationship between age of onset and audio ratings during the Audiovisual-Incongruent condition. (A) Interval size ratings in the A condition. (B) Interval size ratings in the V condition. (C) Interval size ratings in the C condition. (D) Interval size ratings for auditory stimuli in the I condition. (E) Interval size ratings for visual stimuli in the I condition. (F) The relationship between correlation scores between ratings and auditory stimuli in the I condition (AV-I:audio scores) and age of onset of musical training (r = -0.389, p = 0.031). Filled diamonds represent musically trained subjects and open squares represent those who report no musical training.

In the I condition, participants’ ratings were strongly correlated with the audio information, as shown by a significant AV-I:A score, which is the correlation between actual auditory interval size and interval size ratings during the incongruent condition (*r* = 0.850, *p* < 0.0001), but not a significant AV-I:V score (correlation between visual interval size and ratings for the incongruent condition: *r* = -0.231, *n*.*s*.) (Fig 1). This suggests that when confronted with conflicting audiovisual information, participants tended to rely on auditory cues rather than visual cues to make judgments of interval size.

We further explored how individual differences in pitch perception, as moderated by musical training, age, and nonverbal IQ, might affect the degree to which participants varied in their reliance on auditory stimuli when presented with conflicting audiovisual information. We computed pairwise correlations with the variables of AV-I:A scores, age, Shipley abstract scores, and log-transformed pitch perception thresholds (Perc_log). The AV-I:A scores were not significantly correlated with age (*r*(55) = 0.15, n.s.), but showed a significant negative correlation with Perc_log (*r*(55) = -0.333, p = 0.011). This negative correlation indicates that people who were better at pitch perception (lower pitch perception thresholds) were also more likely to rely on auditory cues during incongruent trials. The AV-I:A scores were also significantly correlated with Shipley abstract scores (*r*(55) = 0.537, p < 0.01), suggesting that people who had higher IQ scores were also more likely to rely on auditory cues.

To investigate the effects of age of onset and length of musical training, participants were broken down into the musically trained (n = 32) and musically untrained (n = 25) groups. Participants with musical training showed higher AV-I:A scores (M = 0.92, SD = 0.070) than those without musical training (M = 0.76, SD = 0.39). To compare subjects with and without musical training in AV-I:A scores, a t-test done on AV-I:A scores comparing musically trained and untrained groups showed a significant difference between musicians and non-musicians (t(55) = 2.2, p = 0.034), confirming that musically trained subjects relied more heavily on auditory cues during the audiovisual incongruent condition.

**Fig 2:**
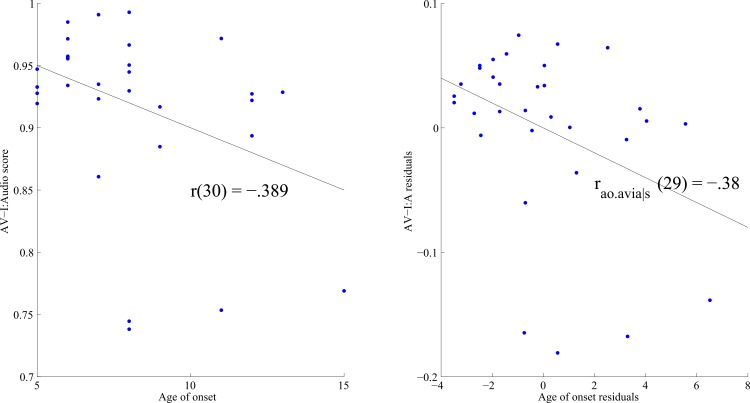
(A) The relationship between age of onset of musical training and AV-I:audio scores for musically trained subjects. (B) The relationship between age of onset of musical training and AV-I:audio scores while partialling out the effect of Shipley scores.

Among people without musical training, AV-I:A scores also showed a significant positive correlation with Shipley abstract score (*r*(23) = 0.596, p = 0.002), showing that subjects with higher nonverbal IQ made ratings that were more correlated with auditory cues when given the incongruent stimuli. Among people with musical training, AV-I:A scores showed a significant negative correlation with age of onset of musical training (*r*(30) = -0.389, p = 0.028; see Fig 2A), indicating that musically trained subjects with an earlier onset of musical training showed higher correlation with auditory cues during the audiovisual incongruent task. Re-calculating the correlation between age of onset and AV-I:A scores for musically trained subjects, without the subjects for whom the AV-I:A score is less than .75 (based on automatic outlier removal rule of 1.5 times the interquartile range below the 25^th^ percentile [22]), resulted in eliminating two more subjects as outliers. Results without these outliers showed an even stronger correlation between age of onset and AV-I:A score: r(28) = -0.515, p = .004. As shown in Table 1, Perc_log was also correlated with Shipley abstract score (r(30) = -.352, p = 0.048), and age of onset was inversely correlated with length of training (r(30) = -.551, p = 0.001), consistent with previous results [23].

**Table 1:** Pairwise correlations between AV-I audio score and Shipley abstraction (nonverbal IQ estimate), log-transformed perception threshold, age of onset of musical training, and number of years of musical training. * Correlation is significant at the 0.05 level (2-tailed). ** Correlation is significant at the 0.01 level (2-tailed).

Correlations	MusicalTraining: Y					
		AV-I audio score	Shipley abstraction	Perc_log	Age of onset	Length of Training
AV-I audio score	r	1	0.102	0.127	-.389*	0.185
	p (2-tailed)		0.578	0.49	0.028	0.309
	df		30	30	30	30
Shipley abstraction	r		1	-.352*	-0.154	-0.029
	p (2-tailed)			0.048	0.399	0.874
	df			30	30	30
Perc_log	r			1	0.333	-0.255
	p (2-tailed)				0.063	0.159
	df				30	30
Age of onset	r				1	-.551**
	p (2-tailed)					0.001
	df					30

** Correlation is significant at the 0.01 level (2-tailed).

* Correlation is significant at the 0.05 level (2-tailed).

Among the subjects with musical training, partial correlations were further conducted to assess the relationship between AV-I:A scores and perception threshold, age of onset of musical training, and length of musical training, while controlling for differences in Shipley abstract score (see Table 2). When Shipley abstract score was controlled in a partial correlation, Perc_log was no longer significantly correlated with the AV-I:A scores (r _p_._avia|s_ (29) = -0.105, p = 0.945). In contrast, age of onset remained significantly correlated with AV-I audio scores even after controlling for Shipley score (r _ao_._avia|s_(29) = -0.38, p = 0.031, Fig 2B). This result corroborates the above analysis that individuals with an earlier onset of musical training rely on auditory cues during this audiovisual task. Length of training and age of onset of training remained significantly inversely correlated, but length of training was not correlated with AV-I:A scores.

**Table 2:** Partial correlations between AV-I audio score and age of onset of musical training, log-transformed pitch perception threshold, and number of years of musical training. * Correlation is significant at the 0.05 level (2-tailed).

Correlations	MusicalTraining: Y					
Control Variables			AV-I audio score	Age of onset	Perc_log	Length of Training
Shipley abstraction	AV-I audio score	r	1	-0.38*	0.174	0.19
		p (2-tailed)		0.035	0.348	0.307
		df		29	29	29
	Age of onset	r		1	0.301	-0.562**
		p (2-tailed)			0.1	0.001
		df			29	29
	Perc_log	r			1	-0.283
		p (2-tailed)				0.123
		df				29

** Correlation is significant at the 0.01 level (2-tailed).

* Correlation is significant at the 0.05 level (2-tailed).

Finally, multiple regression was conducted using the AV-I:A scores as the outcome variable, and Perc_log, Shipley, age of onset, and number of years of musical training as predictor variables. This was done using a stepwise regression in which the order of factors added were: Perc_log, Shipley, Age of onset, and Length of training. Of all the predictor variables, Perc_log, Shipley, and number of years of musical training were not significant; only age of onset emerged as a significant predictor of AV-I:A scores (F(1,22) = 5.603, p = 0.027, age of onset ß = -0.451). Forward and backward regression yielded the same results. Age of onset was the only factor in the final model, further supporting that age of onset of musical training is a reliable predictor of the use of auditory cues in this audiovisual task.

## Discussion

In the present study, we asked how individual differences in pitch perception might affect audiovisual processing, as it relates to factors including years of musical training and age of onset of musical training. Our results show that individual differences exist among strategies used to resolve audiovisual incongruities. When presented with information from only one modality (visual or auditory), ratings of interval size were highly correlated with actual interval size of the stimulus, though performance was better in the auditory domain than the visual domain. Additionally, when presented with visual and auditory stimuli that were congruent and matched, ratings were highly consistent with the actual interval size. Difference scores between congruent and audio-only ratings suggested that while subjects as a group did not use visual information when auditory information was already present during audiovisual congruent conditions, some subjects, in particular those with higher Shipley scores, did appear to use visual information as their ratings were even more accurate (i.e. more correlated with the stimulus) when the stimulus was audiovisual. Furthermore, our results show that while subjects generally relied on auditory information to make their ratings during audiovisual mismatches, the degree to which they relied on auditory information was higher in those with musical training. This degree of correlation with auditory information was only partially dependent on individual differences in pitch perception and nonverbal IQ, but was strongly dependent on the age of onset of musical training. Specifically, the earlier a subject began his or her musical training (if at all), the more likely the subject would use auditory information when confronted with audiovisually incongruent stimuli.

Pairwise correlations showed that individual differences in pitch perception were significantly correlated with audiovisual processing: subjects with superior performance in pitch perception were more likely to rely on auditory cues when given audiovisually incongruent stimuli. However, this effect was not over and above the contribution of IQ differences, whereas age of onset of musical training predicted individual differences in the use of auditory information to solve tasks related to interval size judgment. Thus, while pitch perception and nonverbal IQ do influence the extent to which individuals use auditory stimuli when resolving audiovisual incongruences, our analyses reveal that early musical training remains as the most important predictor of reliance on auditory cues. These results may suggest that individuals with earlier musical training are able to judge the visual stimuli as a distraction and ignore this incongruent information, thereby preferentially prioritizing auditory information when presented with incongruency. How such a mechanism occurs presents a new avenue of research.

These results raise the important question of why early training matters in pitch interval estimation tasks and in audiovisual perception as a whole. One possible mechanism is that a more refined auditory-motor network gives rise to more accurate mental representations, or internal simulations of motor mapping required to produce the interval given the auditory information alone. Musical training is known to provide auditory feedback, thus strengthening sensorimotor associations via the coherence and/or connectivity of auditory and motor systems [24–27]. The stronger auditory-motor network in early-trained musicians could have led to more reliance on the auditory percept in the subjects’ internal simulations of interval size, leading to stronger reliance on auditory information accompanied by relative ease in ignoring potentially distracting visual information among early-trained musicians when facing audiovisual incongruent stimuli. With the present correlational dataset, an alternative explanation that cannot be ruled out is that those who are more inclined to use the auditory modality are more likely to seek out musical training and/or more likely to start earlier in life. Thus, the differences observed between the trained and untrained individuals may not be a result of training but may instead reflect an inherent modality bias that results in some individuals undertaking musical training early in life and others not. While the current dataset cannot tease apart these causal relationships, future longitudinal work may shed light on this problem by defining a longitudinal trajectory of audiovisual processing and musical training.

Taken together, the results show that audiovisual perception is related to musical training. Although early music training is also associated with superior pitch discrimination skills, and superior pitch discrimination is associated with reliance on auditory information in audiovisual perception, our results suggest that early music training plays a critical role in resolving audiovisual incongruence by increasing the subjects’ tendency to rely on the auditory modality. Future studies might test the audiovisual integration of children with different ages of onset of musical training. Understanding how audiovisual processing is affected by the developmental trajectory of musical training may inform our knowledge of neuroplasticity and applications in cognitive training and rehabilitation, while highlighting the complex and eclectic effects of musical training on cognitive and perceptual behavior.
